# Testing of female reproductive disorders

**DOI:** 10.1007/s00204-020-02883-3

**Published:** 2020-08-24

**Authors:** Hermann M. Bolt

**Affiliations:** Leibniz Research Centre for Working Environment and Human Factors, Ardeystr. 67, 44139 Dortmund, Germany

Recently, Johansson and colleagues published an article about the mechanisms causing female reproductive disorders with a special focus on the ovaries (Johansson et al. 2020). Disturbed ovarian development is referred to as the ovarian dysgenesis syndrome (Buck Louis et al. 2011; Johansson et al. 2017) and a discussion is ongoing if and to which degree environmental factors may contribute (Fowler et al. 2012; Hunt et al. 2016; Johansson et al. 2017). In the present study, the authors established several adverse outcome pathways (AOPs) using the AOP standard terminology (Leist et al. 2017; Vinken et al. 2017). According to Johansson et al., the most relevant AOPs are (1) aromatase (Cyp19a1) reduction, (2) disrupted meiosis, (3) ectopic estradiol or AHR activation, (4) disrupted folliculogenesis and (5) disrupted follicle maturation. The practical relevance is that key events of these AOPs can be tested in cell culture, thereby linking an adverse outcome in humans to in vitro testable readouts.

In recent years, stem cell-based tests have been established where precursor cells are exposed to test compounds during the differentiation period (Krug et al. 2013; Waldmann et al. 2014; 2017; Zimmer et al. 2014). Based on in vitro gene expression data, it was possible to identify developmental toxicants and to differentiate them from negative control compounds (Balmer et al. 2014; Weng et al. 2014; Shinde et al. 2015; 2016, 2017). However, in vitro systems of the ovary that reliably represent the human situation still remain to be established and validated. A further challenge to be addressed is scaling, i.e., to identify if—and if yes by which factor—higher concentrations have to be used in vitro compared to the plasma peak concentrations in vivo (Albrecht et al., 2019; Godoy et al., 2013). Although the recently published AOPs of Johansson and colleagues (2020) are an important step towards a more systematic analysis of female reproductive disorders, there is still a long way to go until cause–effect relationships between chemical exposure and reproductive disorders can be reliably established.

## References

[CR1] Albrecht W, Kappenberg F, Brecklinghaus T, Stoeber R, Marchan R, Zhang M (2019). Prediction of human drug-induced liver injury (DILI) in relation to oral doses and blood concentrations. Arch Toxicol.

[CR2] Balmer NV, Klima S, Rempel E, Ivanova VN, Kolde R, Weng MK (2014). From transient transcriptome responses to disturbed neurodevelopment: role of histone acetylation and methylation as epigenetic switch between reversible and irreversible drug effects. Arch Toxicol.

[CR3] Buck Louis GM, Cooney MA, Peterson CM (2011). The ovarian dysgenesis syndrome. J Dev Orig Health Dis.

[CR4] Fowler PA, Bellingham M, Sinclair KD, Evans NP, Pocar P, Fischer B (2012). Impact of endocrine-disrupting compounds (EDCs) on female reproductive health. Mol Cell Endocrinol.

[CR5] Godoy P, Hewitt NJ, Albrecht U, Andersen ME, Ansari N, Bhattacharya S (2013). Recent advances in 2D and 3D in vitro systems using primary hepatocytes, alternative hepatocyte sources and non-parenchymal liver cells and their use in investigating mechanisms of hepatotoxicity, cell signaling and ADME. Arch Toxicol.

[CR6] Hunt PA, Sathyanarayana S, Fowler PA, Trasande L (2016). Female reproductive disorders, diseases, and costs of exposure to endocrine disrupting chemicals in the European Union. J Clin Endocrinol Metab.

[CR7] Johansson HKL, Svingen T, Fowler PA, Vinggaard AM, Boberg J (2017). Environmental influences on ovarian dysgenesis: developmental windows sensitive to chemical exposures. Nat Rev Endocrinol.

[CR8] Johansson HKL, Damdimopoulou P, van Duursen MBM, Boberg J, Franssen D, de Cock M (2020). Putative adverse outcome pathways for female reproductive disorders to improve testing and regulation of chemicals. Arch Toxicol.

[CR9] Krug AK, Kolde R, Gaspar JA, Rempel E, Balmer NV, Meganathan K (2013). Human embryonic stem cell-derived test systems for developmental neurotoxicity: a transcriptomics approach. Arch Toxicol.

[CR10] Leist M, Ghallab A, Graepel R, Marchan R, Hassan R, Bennekou SH (2017). Adverse outcome pathways: opportunities, limitations and open questions. Arch Toxicol.

[CR11] Shinde V, Stöber R, Nemade H, Sotiriadou I, Hescheler J, Hengstler J, Sachinidis A (2015). Transcriptomics of hepatocytes treated with toxicants for investigating molecular mechanisms underlying hepatotoxicity. Methods Mol Biol.

[CR12] Shinde V, Perumal Srinivasan S, Henry M, Rotshteyn T, Hescheler J, Rahnenführer J (2016). Comparison of a teratogenic transcriptome-based predictive test based on human embryonic versus inducible pluripotent stem cells. Stem Cell Res Ther.

[CR13] Shinde V, Hoelting L, Srinivasan SP, Meisig J, Meganathan K, Jagtap S (2017). Definition of transcriptome-based indices for quantitative characterization of chemically disturbed stem cell development: introduction of the STOP-Toxukn and STOP-Toxukk tests. Arch Toxicol.

[CR14] Vinken M, Knapen D, Vergauwen L, Hengstler JG, Angrish M, Whelan M (2017). Adverse outcome pathways: a concise introduction for toxicologists. Arch Toxicol.

[CR15] Waldmann T, Rempel E, Balmer NV, König A, Kolde R, Gaspar JA (2014). Design principles of concentration-dependent transcriptome deviations in drug-exposed differentiating stem cells. Chem Res Toxicol.

[CR16] Waldmann T, Grinberg M, König A, Rempel E, Schildknecht S, Henry M (2017). Stem cell transcriptome responses and corresponding biomarkers that indicate the transition from adaptive responses to cytotoxicity. Chem Res Toxicol.

[CR17] Weng MK, Natarajan K, Scholz D, Ivanova VN, Sachinidis A, Hengstler JG (2014). Lineage-specific regulation of epigenetic modifier genes in human liver and brain. PLoS ONE.

[CR18] Zimmer B, Pallocca G, Dreser N, Foerster S, Waldmann T, Westerhout J (2014). Profiling of drugs and environmental chemicals for functional impairment of neural crest migration in a novel stem cell-based test battery. Arch Toxicol.

